# Sustainable improvement and evaluation of the shifting smoothness of vehicle transmission

**DOI:** 10.1038/s41598-021-02130-4

**Published:** 2021-11-19

**Authors:** Yongxiang Li, Chujin Hu, Zhenwen Chen, Chunhui Wang, Jing Li, Haixia Guo

**Affiliations:** 1 Hangzhou Vocational and Technical College, Hangzhou, 310018 China; 2 Xingzhi College, Zhejiang Normal University, Jinhua, 321004 China; 3 Zhejiang Wanliyang Co. Ltd, Jinhua, 321025 China

**Keywords:** Sustainability, Mechanical engineering, Energy efficiency

## Abstract

Energy savings and environmental protection are the only way for the sustainable development of the automotive industry. The poor shifting performance of automobile transmission may reduce the driver's driving pleasure and make the driver feel tired. In addition, improper shifting would also increase fuel consumption. Therefore, in view of the importance of improving the shift performance of vehicle transmission, GSA testing technology was employed for the existing shift quality problems of commercial vehicles to continuously realize high shifting efficiency and low fuel consumption. Through the establishment of subjective and objective evaluation criteria of the experimentally determined shift performance of commercial vehicles, a reliable theoretical basis is provided for product optimization design and shift performance evaluation. As a result, the shift control strategy and optimization matching measures are formulated to ensure that the power, smoothness and transmission of the whole vehicle system meet the technical requirements and finally achieve a rapid and stable gearshift. Thus, this work unveils the high potential of improving the shift performance and quality of the whole vehicle and is expected to have an impact on reducing fuel consumption and emissions in the relevant automotive industry, contributing to the establishment of a more sustainable society.

## Introduction

Today, we are facing the cruel reality that the global oil reserves are decreasing, while the global oil consumption is increasing year by year. This directly cause the supply and demand situation of global crude oil to become not optimistic. Moreover, with the rapid growth of global vehicle ownership and the increasing pressure brought by energy and the environment, it is more urgent for the automotive industry to solve the problems of energy and pollution in order to achieve sustainable development^1–3^. Under the development trend of "green, energy saving and environmental protection" in the automobile industry, we recently proposed a novel approach for the in-depth study of vehicle energy-saving technology from the aspect of the gear shifting strategy control, which is conducive to environmental protection and energy security all over the world, so as to realize the sustainable development among human, resources and the environment in the field of the automobile industry.

At present, the mainstream vehicle energy-saving paths mainly include miniaturization, lightweight, low friction, diesel passenger cars, efficient power transmission systems (internal combustion engines, gearboxes), hybrid vehicles and so on^4^. An overview of the energy-saving technology routes of European Union and Japan, combined with the basis and characteristics of China's energy-saving technology, helps us have an overall concept. That is, the high-efficiency powertrain technology and lightweight vehicles should be the key development directions of China's energy-saving technology in the near future^5–7^. Among them, the high-efficiency powertrain technology is widely regarded as the most important and effective way to improve the fuel economy of vehicles, which is mainly due to the loss of more than 72% of the energy generated by vehicle fuel combustion through the exhaust, heat transfer and mechanical friction of the powertrain^8^. Therefore, how to effectively improve the efficiency of power transmission systems is an urgent task for the development of energy-saving technology.

In the foregoing vehicle powertrain, the vehicle transmission is the core component of the vehicle integrated driving control system and has an important impact on the energy consumption of the whole vehicle^9^. It changes the speed and torque through manually adjusting the gear meshing relationship in the transmission by the control lever and shift fork, which could realize the required change of transmission ratio, help to make the transmission system best match with the engine working condition, and improve the fuel economy and power performance of the whole vehicle. Then, how to achieve the balance between economy, power, drivability and NVH in the design and matching of vehicle transmission is not only about the transmission itself, but also needs to be considered in the whole transmission chain. Within this context, the system testing platform based on GSA proposed in this paper is just for such requirements in the implementation of the vehicle shift evaluation. It aims to further reduce the energy consumption and emissions of vehicles through the newly developed testing system for vehicle shifting performance based on GSA testing technology, to enhance the competitiveness of the vehicle powertrain in power performance, fuel economy, comfort and other aspects.

This study employed the literature analysis to show a detailed overview of the current knowledge concerning the positive impact of shifting performance on the vehicle energy-saving research and green driving. With the increasing demand for vehicle handling comfort and energy conservation and environmental protection in the market, developing the reliable shift testing system for vehicle transmission has become one of the keys to improve the competitiveness of vehicle products^10^. The study found that uncomfortable and unsmooth driving interferes with the association between fuel consumption, emissions and driver personalities^11^. Poor vehicle transmission performance will reduce the driver's driving pleasure, make the driver feel tired, increase fuel consumption, and affect the driver's green driving. Especially in urban traffic conditions, the driver is very concerned about the comfort and smoothness of gear selection and shifting performance because the vehicle is always continuously engaged in gear selection and shifting action without interruption, and the performance index will directly affect the driver's efficiency assessment on a certain type of traffic vehicle^12,13^. It can be said that as an easy perception item for users, the shift performance of the vehicle transmission has a direct relationship with the degree of user complaints. The smoothness of vehicle shifting can not only ensure green driving but also prolong the service life of the vehicle, which is more conducive to environmental protection^14^. Therefore, to meet the needs of consumers, as well as the requirements of energy savings and environmental protection, vehicle manufacturers need to continuously optimize the driving shifting mechanism to improve the shifting quality as much as possible in the process of transmission development and matching to reduce fuel consumption and improve driving comfort^15^. As a result, the innovation and contributions of the present study is to strive for optimizing the shift efficiency in vehicle powertrain to create sustainable value and economic growth.

In view of the above, much effort has been directed at the development of safe, reliable and competitive methods for testing and evaluating the shifting performance of vehicle transmission. In the early days, the shifting evaluation test of vehicle transmission mainly adopted two modes, including road tests and bench tests^16^. Because road tests are greatly affected by natural factors, more importantly, the test cycle is too long and the cost is too high, so it is an inevitable trend to develop bench tests for transmission shift performance. In the boom of the rapid development and wide application of virtual instrument and sensor technologies, the bench test of gearshift performance of vehicle transmission, with the characteristics of high accuracy and high integration, has gradually become the main testing method^17,18^. It involves the hardware platform of the test bench and the software design for measuring and controlling the system. The testing data mainly include shift force and shift stroke. The curve of the shift force and displacement can also be obtained by offline analysis to evaluate the speed change. In particular, the comprehensive test bed developed by Volkswagen in 1991 is quite amazing, and it can simultaneously carry out life tests of transmission, clutches, transmission shafts and rear axles^19^. However, the disadvantage of the shifting test bench is that it is difficult to avoid the negative impacts of artificial manipulation. There is a lack of a simulation environment for the whole vehicle system, especially for the detailed synchronizer model, which leads to not only low efficiency but also poor cost performance^20^. In addition, the difference in personal operating habits and subjective feelings would also interfere with the objectivity of the test data, which is not convenient for the evaluation of the shifting performance of vehicle transmission.

Up to now, there are mainly two ways for shift evaluation test of vehicle transmission: subjective evaluation test and objective evaluation test. The objective evaluation test is subdivided into static evaluation test and dynamic evaluation test^21^. Subjective evaluation test refers to the experienced drivers are recommended to make subjective test scores on various gear shifting performances when the vehicle is stationary or running, and the subjective comprehensive score of various performances is taken as the evaluation test index^22^. The level of subjective evaluation test depends on the long-term personal experience of the test driver, which would have obvious deficiencies such as too strong subjectivity, great influence of personal factors, and inability to quantify specific test indicators, as well as the information transmission between the driver and the designer is not accurate enough. Hence, the objective evaluation test is more in line with the actual shift operation of vehicle transmission and is favored. Static evaluation test is carried out under the conditions of normal temperature, vehicle stationarity, engine off and clutch separation^23^. Although static evaluation test is very common in transmission manufacturers and is also the primary means of evaluating the gear shifting performance, the clutch is in the disengaged state, resulting in the transmission gear being at rest during the measurement, which has no effect on the shifting. However, customers always perform the shifting operation when the vehicle is running and the transmission is working. Therefore, it is relatively difficult for the static evaluation test to reflect the shift performance under the vehicle running state, which is different from the customer's use condition.

**Figure 1:**
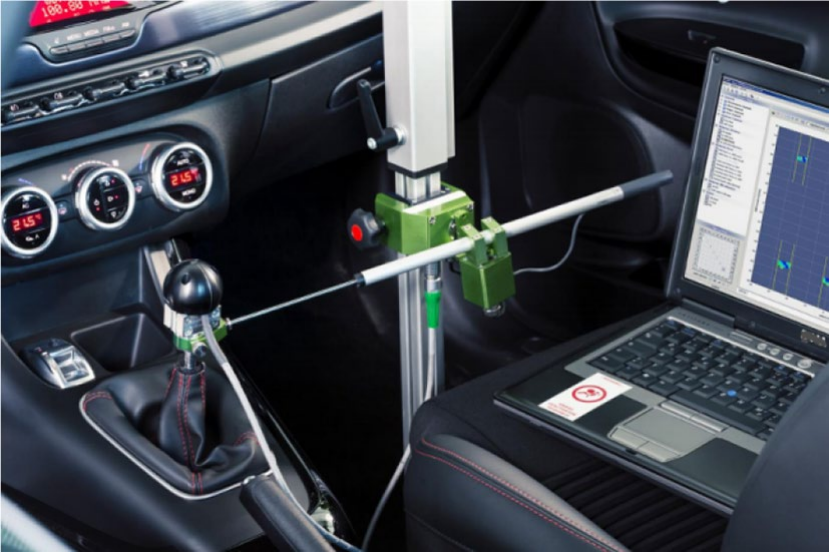
Shift testing system of the whole vehicle.

**Figure 2:**
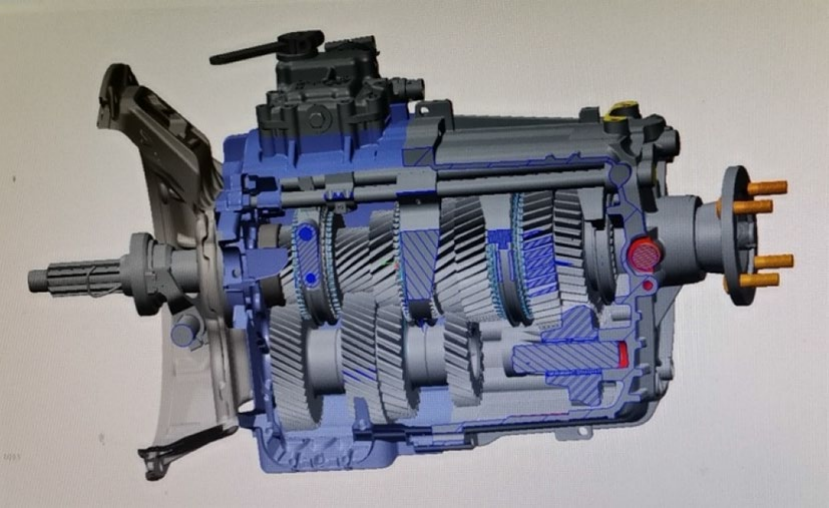
Schematic diagram of the analyzed transmission.

In the present study, aiming at the limitations of the above cited literatures in the subjective and static shifting evaluation tests of the existing vehicles, we aim to adopt a new type of GSA (gear shift analysis) testing technology to explore the dynamic evaluation test and the subsequent corrective measures for the dynamic shifting performance of vehicle transmission, which has filled the vacancy of previous studies. As shown in Fig. 1, the whole GSA experimental device involves three-axial load sensor, three-axial displacement sensor, travel sensor, rotational speed sensor, temperature sensor (K type), digital thermometer, measurement arm (tripod), IMC measurement device, wires, pushbutton device, TTL converter, etc. Among them, it should be pointed out that GSA high quality force knob sensor is used to acquire high accuracy force values in shift, select and vertical direction, and GSA travel measurement unit is used to acquire shift and select travel of the shift knob, and GSA capture software is used to acquire the signals from the sensors and realizes online displays for sensor verification and gear shift counting. The outstanding advantages and innovation of this research are mainly reflected in that the most suitable debugging options could be adjusted through the data reflection on GSA analysis software, which is applied to a commercial vehicle with manual transmission (as shown in Fig. 2) for objective testing of shifting performance. Herein, combined with past subjective test evaluation experience, the comprehensive analysis between objective and subjective evaluations of shifting performance could assist designers in systematically and comprehensively evaluating the shifting performance of vehicle transmission at each stage of the product life cycle. Using the objective test to explain the subjective phenomenon could more intuitively determine the difference of the numerical curves under various working conditions, debug the shifting parameter values reasonably to achieve the purpose of improving the shifting performance systematically, and then improve the driving comfort and energy efficiency of the vehicle. Finally, this study would effectively fill the technical gap in the objective dynamic shifting evaluation test of vehicle transmission, and make the test technology play an increasingly important role in improving the shifting efficiency of commercial vehicles, which would be conducive to the energy conservation and emission reduction of vehicles to promise a sustainable future.

## Methodology

With the continuous popularization of commercial vehicles in the market and the deepening of consumers' awareness of driving comfort, people pay increasing attention to the overall shift quality of vehicles. Especially in terms of environmental protection and energy savings, the shifting efficiency has become an important aspect that major vehicle transmission manufacturers strive to improve, and then the corresponding assessment of vehicle shift performance is also more detailed and in-depth^24,25^. GSA testing technology could quantify the subjective feelings of drivers into objective data, which could guide the formulation of improvement measures. In view of its incomparable advantages, this new type of testing technology has very broad application prospects and high research value. Usually, the whole testing process could be described as follows: First, the whole GSA acquisition installation components would be orderly placed in the test prototype to ensure that all components can move smoothly in the working motion range without interference; second, the vehicle would be started, the test prototype would be powered on, and GSA hardware acquisition equipment would be opened and carefully debugged. After the whole system is in a normal state, the data acquisition instrument begins to follow the tracks of the test data during the shift operation. Again, considering that the above testing data could not be automatically identified, we also need to use the acquisition software to convert the gathered analog signal into directly identifiable information for subsequent operation. Finally, GSA software analyzer, according to the above converted information, would perform the analysis and evaluation of the shifting characteristics and then give the testing analysis reports and guidance schemes.

**Figure 3:**
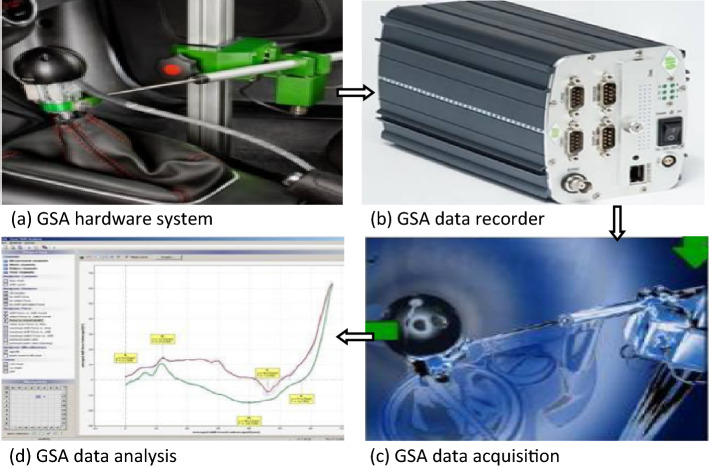
Shift testing and analysis process.

This study draws support from the establishment of GSA shifting test and evaluation system to realize research on the control strategy of vehicle shifting quality. The proposed technical route is represented in Fig. 3. Generally, the first step is to formulate a detailed research programme and form a theoretical research framework oriented to the vehicle shifting test and evaluation. Furthermore, by collecting relevant product data and summarizing the technical parameters of vehicle transmission and its steering mechanism, we could preliminarily predict the development status of the whole shifting system. Then, preparations are started to set up GSA testing system for reliable shifting control performance. It is necessary to perform the subjective and objective evaluation analysis of the shifting process, and after real-time collection, analysis and evaluation of various performance indexes in the shifting process, it would provide data and theoretical support for systematically improving the shifting performance. In a further step, the specific optimization solutions of the vehicle shifting mechanism are put forward, and the shifting performance of the vehicle is retested and evaluated again to obtain the optimum matching parameters of the shifting mechanism and then experimentally verify the effectiveness of the proposed improvement measures. Finally, the testing optimization path for the shifting performance based on GSA testing technology is established.

It should be noted that GSA testing system consists of a hardware acquisition device and software performance analysis tools. The system realizes the remote collection, monitoring and treatment of the testing data of the running shift mechanism, such as force, stroke and acceleration of the swing arm end of the shift knob. The shift performance of the whole vehicle is systematically evaluated from the main index items, such as shift force (stroke), system stiffness, free play and dynamic shift. The comprehensive gear selection and shifting performance of the whole vehicle is estimated from the main index items, such as selection and shift force (stroke), system stiffness, free clearance and dynamic shift. In view of some existing problems of gear shifting performance, GSA testing analysis is carried out on the testing vehicle with the transmission assembly.

### Gear shifting force

**Figure 4:**
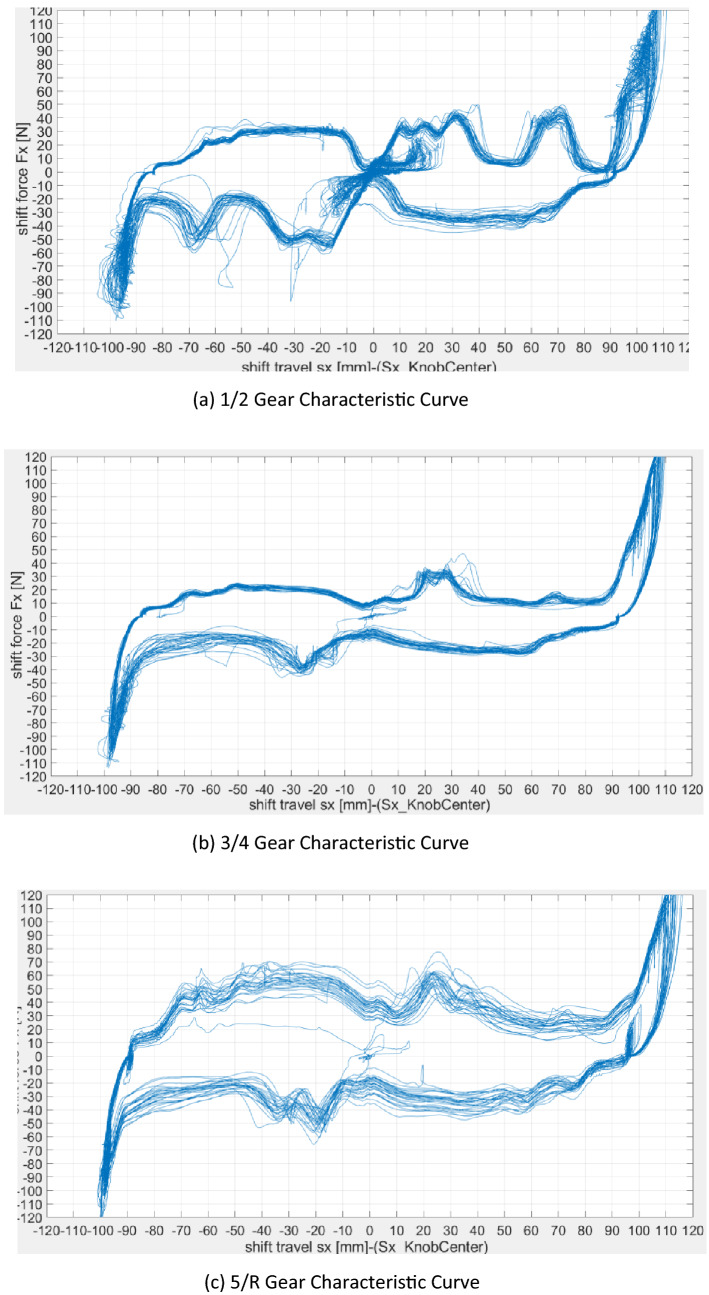
The characteristic curve of different gears.

The Detent module of GSA is used to test the vehicle's gear shifting performance. The practical shifting force curve is represented in Fig. 4. The conclusive posttest data information is described in Table 1.

- The shifting force/pulling force of each gearshift position is appropriate
- The characteristic curve of the 5/R gear is divergent, which is caused by the overspeed shift structure of the 5/R gear but does not affect the actual use.
- Inhalation sensation of the gearshift position was not evident.

**Table 1:** Shifting performance comparison data of three gears.

	The shift performance test data						
	Item	Test gear					
		N-1-N	N-2-N	N-3-N	N-4-N	N-5-N	N-R-N
Transmission assembly model	Maximum shifting force	43.6	50.4	33.36	38.8	57.6	50.5
	Maximum pulling force	33.1	32.3	26.3	22.7	37.7	57.9
	Shifting traverses	92	83	92	85	95	87
	Inhalation sensation	Not obvious	Not obvious	Not obvious	NOT obvious	Not obvious	Not obvious
	Sense of leaning against the wall	Obvious	Obvious	Obvious	Obvious	Obvious	Obvious

### Gear selecting force

The driver's operation experience and the vehicle's dynamic shifting performance are associated with the functional property of the vehicle's gear selection to a considerable degree. It is better that the slope of the selecting force curve would not be too small but not too large. The main reason is that a slope that is too small would make the free play in the neutral position too large, and if it is too large, the gear selecting force may exceed the applicable range, thus interfering with the normal driving operation of the vehicle. Therefore, the selected force curve should be best developed horizontally or slightly downward.

**Figure 5:**
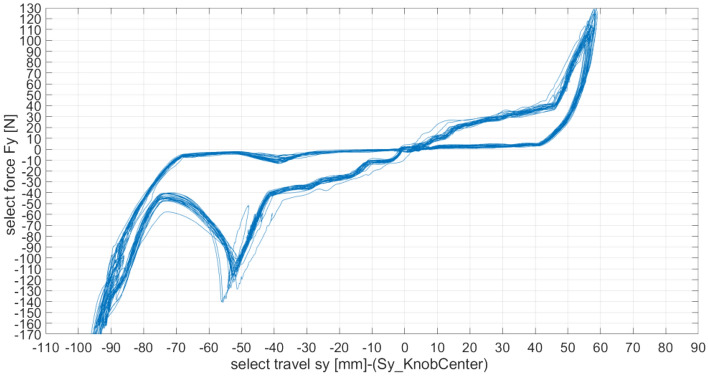
The actual selection performance curve.

The practical selecting force curve is represented in Fig. 5. The conclusive posttest data information is described in Table 2.

- The gear selecting force of each gearshift position is appropriate, and the gear selecting process is relatively level and smooth without sticking.
- The selected journey of each gearshift position is relatively consistent.
- The dividing line of gear selection between the 1/2 gear and R gear appears to be clear, and there is still an evident inhalation sensation of the gearshift position.

**Table 2:** Selecting performance comparison data of three gears.

The selection performance test data				
	Item	Test gear		
		N-1/2	N-3/4	N-5/R
Transmission assembly model	Maximum selecting force-N	40	38	110
	Maximum resilience-N	5	5	5
	Selecting traverses-mm	42	45	75

### Free play and H pattern

The situation of the free play can reflect the accuracy of the gear shift rod, and the proper free play could ensure the credibility of the shifting operation and comfortable driving experience. The function of the H pattern is mainly reflected in that it could plainly depict the boundary between different gearshift positions. Therefore, we could clearly determine the suitability of each gearshift position if the free play and H pattern are analyzed together.

**Figure 6:**
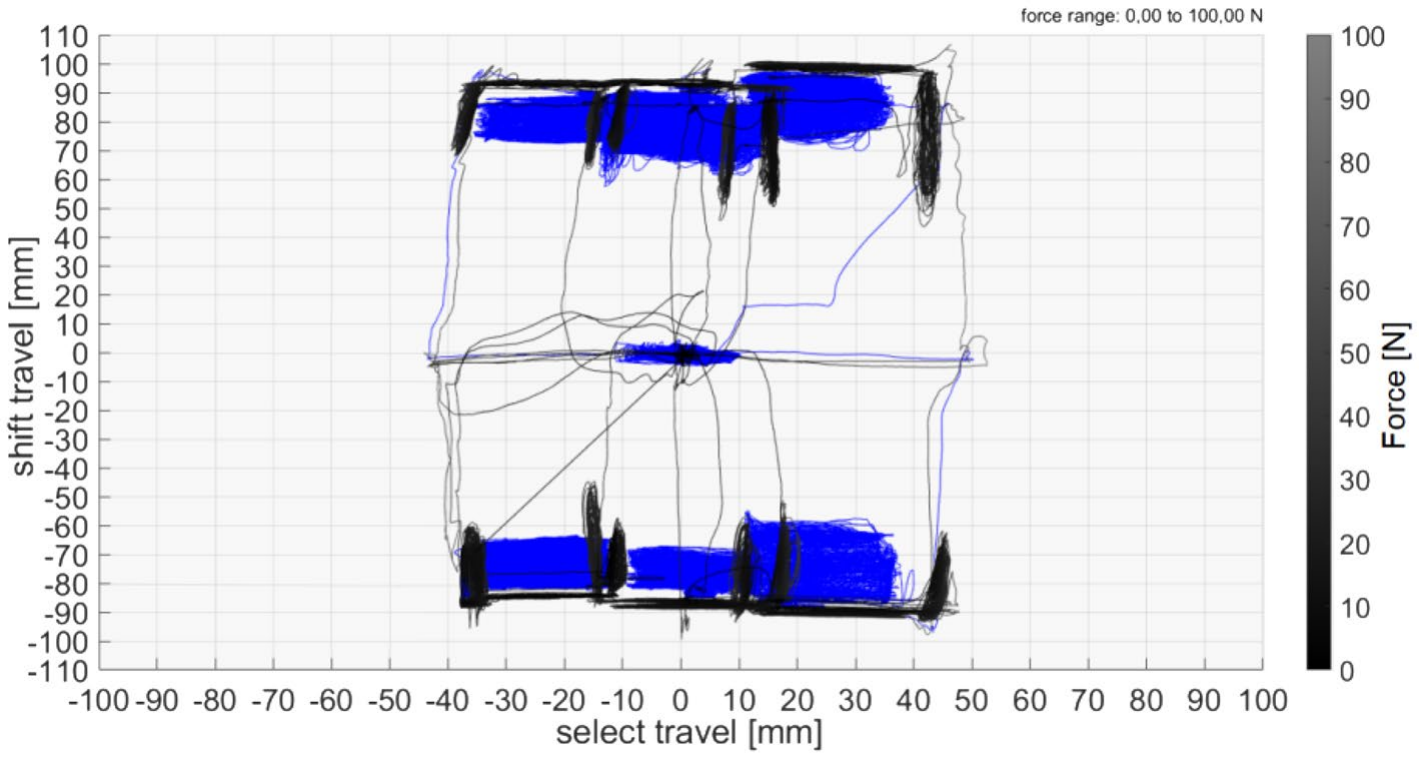
The actual diagram of H pattern and free play.

The practical diagram of the H pattern and Free Play is represented in Fig. 6. The conclusive posttest data information is described in Table 3.

**Table 3:** H-Pattern and free play diagram under 10 N force.

H-pattern and free play diagram under 10 N force mm							
	Test gear						
	N	1	2	3	4	5	R
Transmission assembly model	8 × 20	15 × 20	18 × 25	23 × 25	13 × 20	20 × 20	28 × 25

It could be analyzed and judged from the following table.

- There is a small amount of overlap in the adjoining region, and the whole regional arrangement is in good order.
- The free play of each gearshift position is slightly larger.

### Cross shift

The functionality of cross shifting is an important index to weigh the performance of the whole shift system. It would be decided by the inner structural parameters' optimization design of the executive system and the quality control of the vehicle component.

**Figure 7:**
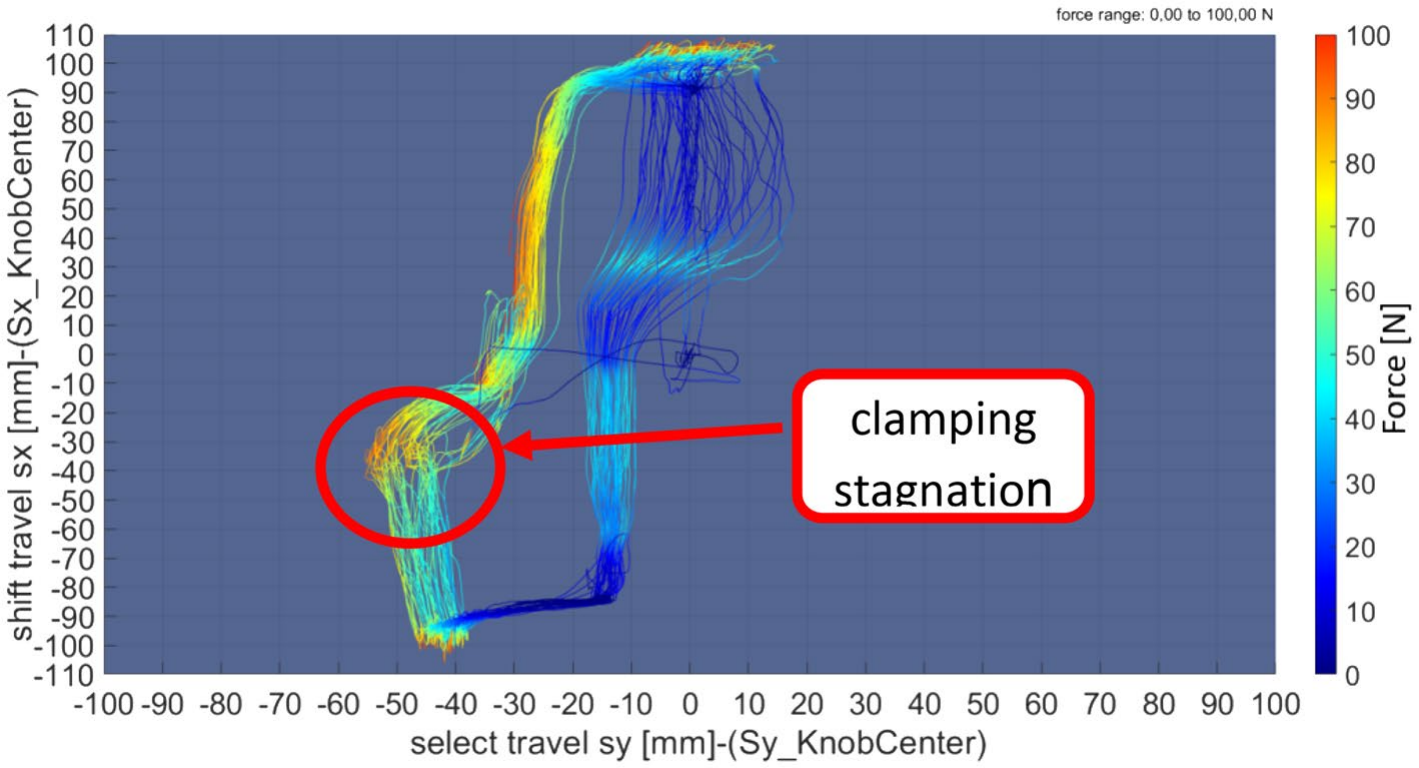
The actual diagram of cross shift (2–3–2).

**Figure 8:**
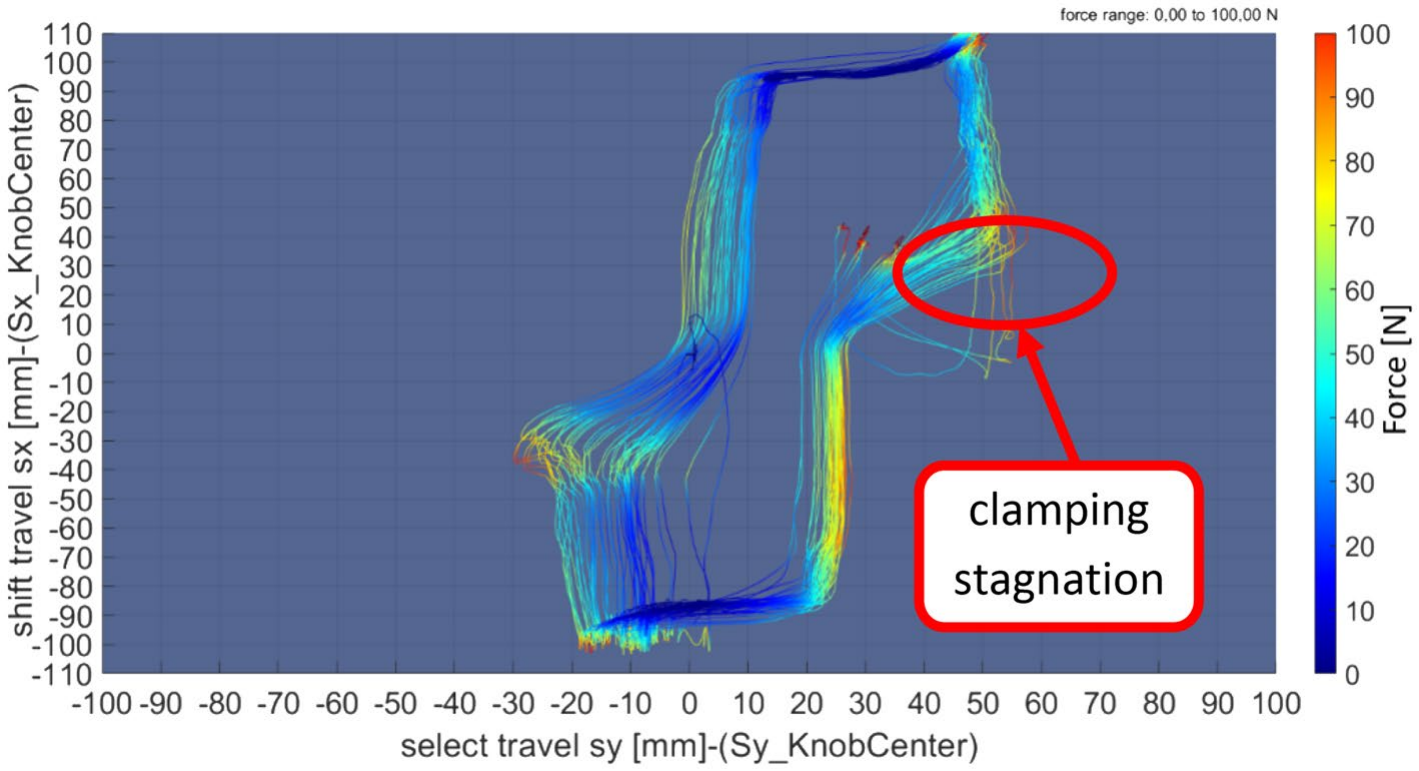
The actual diagram of cross shift (4–5–4).

The practical diagram of the cross shifting is represented in Figs. 7 and 8. It could be analyzed and judged from the following diagram.

- The characteristic curves from gears 2 to 3 are relatively scattered.
- The characteristic curves from gears 5 to 4 are relatively scattered.
- The characteristic curves from gears 4 to 5 show that there is shift clamping stagnation in the process.

### Shift and select rigidity

Shift and select rigidity is another index for evaluating the gear shift operating system. Proper selection of the shift and selection of rigidity could help to weaken the shift clamping and secondary impact. However, if the shift and select rigidity is preset to be too large, the subjective experience of the shift operation would become worse. Therefore, stepwise debugging the feasible shift and selecting rigidity would be particularly critical.

**Figure 9:**
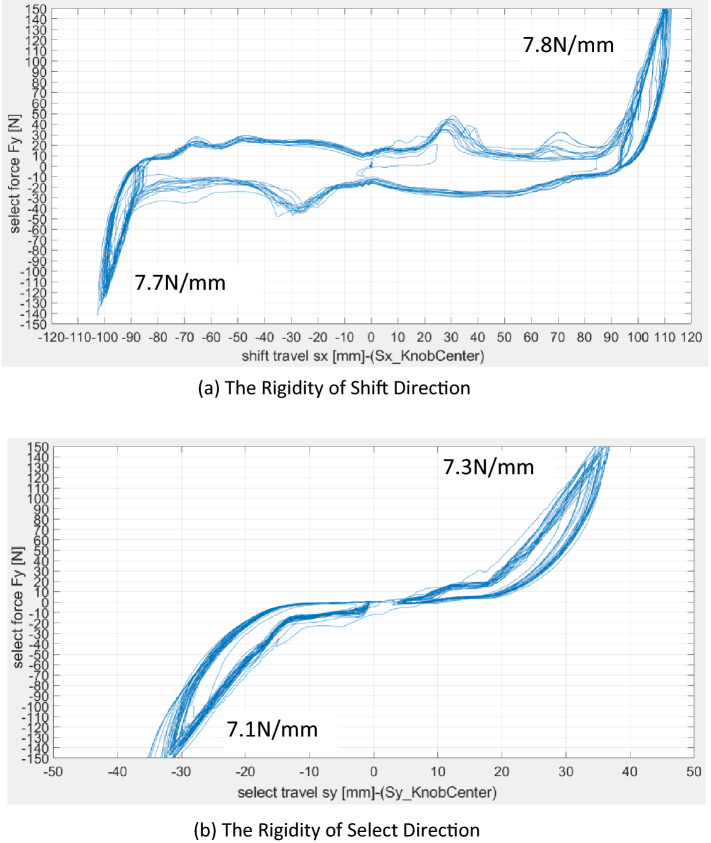
The actual shift and selected rigidity.

The actual shift and selected rigidity are represented in Fig. 9. It could be analyzed and judged from the following figures.

- The recommended shift rigidity given by GIF is generally assumed to be 5 N/mm-8 N/mm, and the original observed values are 7.8 N/mm and 7.7 N/mm. The shift rigidity of the whole test system should be within a reasonable range.
- The recommended selected rigidity given by GIF is generally assumed to be 4 N/mm-6 N/mm, and the original observed values are 7.3 N/mm and 7.1 N/mm. The selected rigidities of the whole test system are too large relative to the reasonable range.

## Results and discussion

In this paper, a GSA testing system is used to test the shifting performance of vehicle transmission. By fitting the testing data, a series of parameter change curves are formed. The subjective evaluation of the shifting performance is objectified and quantified, and the items for improving the shifting performance are directly determined. From the final calculation results and the related real vehicle matching experimental evaluation, there may be the following problems with the shifting performance of the vehicle:

- There is no obvious sense of inhalation during the shifting operation.
- A high possibility of clamping stagnation may occur during the implementation of the cross-shift operation.
- The deformation degree of the whole shifting system is relatively large, which may have exceeded the specified service scope. This would bring an obvious sense of wall collision in the process of gear selection, and the gear selector lever with excessive rigidity could clearly transmit the vibration of the transmission to the ball head for gear selection, which has a significant impact on the accuracy and comfort of the actual gear selection operation.

In view of the above problems, we could take relevant measures to adjust the shifting performance of the vehicle transmission by improving the shifting structure and the related design parameters. Specifically, it can be improved from the following aspects:

- Generally, the shifting inhalation sense is supposed to effectively reduce the distance for the drivers to move the gear lever, and it can also facilitate the relative reduction of the shifting force, which plays a good role in the increase of the shifting feel. Moreover, it could effectively attenuate the vibrational amplitude caused by the gear shaft parts and has a positive effect on the driving comfort of the vehicle. Under this circumstance, we could try to adjust the size of the shift positioning blocks and add proper linear bearings as an effective measure to improve the shifting inhalation sense.
- It is generally believed that the interlocking plate is mainly used to prevent the shifting gears from entering two shifting positions simultaneously. When the structural size of the interlocking plate exhibits abnormal problems, if there is a slight deviation in the gear selection position, the shifting blocks of each shifting position would be seized up and stopped by the interlocking plate. As a result, the shifting head could not move the shifting block, resulting in the shift clamping stagnation phenomenon. Under this circumstance, it could be considered to proceed from the structure adjustment of the interlocking block and the local size modification of the shifting head to eliminate the above clamping phenomenon. The main features of the structure could realize uninterrupted power, high shifting efficiency and low fuel consumption in the process of vehicle shifting.
- The function of the dragline in the gear control system is to transfer the gear action from the driver for the control mechanism to the transmission to realize gear selection and shifting. It is suggested to select the appropriate grease for dragline according to the vehicle type, ensure proper clearance between the mandrel and the protecting tube, and reduce the bending of the dragline at a large angle as much as possible. This could improve the matching degree of the vehicle powertrain, which is conducive to further reducing the energy consumption on the original basis.

In summary, during the design and development of modern vehicle systems, vehicle dynamics control systems play an important role in vehicle safety, comfort, economy and energy efficiency. In this paper, we attempt to discuss the operating principle of GSA testing system at length, highlighting that the novel system tool could not only help engineers analyze the product design of vehicle transmission in a greater degree of freedom, but also improve the efficiency of the whole vehicle integrated drive development process, and then systematically analyze the defects of a certain vehicle in the course of gear shifting by combining with the example of the experimental center of the university where the author works, and finally determine the key elements of the research object that need to be improved. As far as the contribution of its practice and methodology is concerned, the advanced system analysis tools and equipment testing process involved in this paper are very important for the development of innovative vehicle powertrain products, especially under the pressure of increasingly stringent environmental standards, cost control and R & D cycles. With this technology, we could quickly seek out all the key points that should be considered in various elements, through the establishment of objective evaluation, test and analysis of the shift performance of commercial vehicles, and finally make a breakthrough in improving the shifting performance and reducing the shifting force. The shift performance of vehicle transmission assists the sustainable development of automobile industry by bettering transmission efficiency together with increasing fuel utilization. Information gathering and processing improvements of GSA testing technology applied to vehicle shift performance discussed in this study enable better management of vehicle energy efficiency and the reduction via vehicle shift smoothness. This would further help drivers engage in green driving as much as possible to effectively reduce environmental pollution and save energy.

## Conclusions

The sustainable development of the automobile industry plays an important role in ensuring global energy security, coping with climate change and improving the ecological environment. It will also be an important engine to promote the sustainable growth of the global economy in the future. Therefore, promoting the sustainable development of the automobile industry is the common responsibility and goal of stakeholders in the global automobile field. With the increasing maturity and high-end oriented development of the commercial vehicle market, the requirements for the handling comfort of the vehicle are also improved. In particular, for vehicle transmission, as an important core component of the commercial vehicle, its shift performance have a direct impact on the drivability of the whole vehicle. This paper is implemented to clarify an objective evaluation method of vehicle shift performance based on GSA testing technology, which could accurately record the static and dynamic data of vehicle shifts, and then through data processing and analysis, it is convenient for the professionals and practitioners to quickly and accurately find the key parts with some defects and solve them. Its scientific value is mainly reflected in the interpretation of subjective evaluation phenomena with objective test data with the help of GSA testing technology, which could quantify the driver's subjective experience as objective data reports, offer powerful support to improve the shifting performance of vehicle transmission.

This study’s contributions are guiding the formulation of relevant optimization strategies and then enhance the shift quality and efficiency of vehicle transmission, in order to achieve the goal of the vehicle energy-saving transformation, and ultimately improve the power and economy of the vehicle. Meanwhile, this study plays a positive role for the cooperative institutions of vehicle manufacturing enterprises to improve the vehicle shift performance. The related methods and findings would be helpful to further promote the optimal matching between the vehicle components, save considerable time and resources, reduce the number of optimizations and tests in the later stage, shorten the research and development cycle, and provide valuable reference data for the research and development of high-tech products in the later stage of the enterprise. Besides, the research results would also expand its potential influence on the whole automobile industry, offering certain theoretical directions and helpful practical advice for the automobile industry in the debugging and analysis of vehicle shifting performance optimization. Thus, it could make efforts to reduce the environmental burden in the whole life cycle of vehicle transmission systems, realize the win–win situation of environmental and economic benefits for vehicle enterprises, make contributions to mitigating climate change and solving environmental problems, and finally continue to help the construction of a low-carbon society.

There are some limitations in the database available for this study, which would cause that the number and scope of references cited in this study are not adequate, and also include low impact sources, resulting in insufficient research background and affecting the academic impact value of this paper. Therefore, future studies would employ more databases and incorporate different literature sources, to better improve the scientific value added and academic contribution of the follow-up research results in comparative elaboration.

## References

[1] Huang H, Liang DP, Liang L (2019). Research on China's power sustainable transition under progressively levelized power generation cost based on a dynamic integrated generation-transmission planning model. Sustainability.

[2] Fu YW, Chen XM (2019). Thoughts on the law of automobile industry development. Strateg. Study CAE.

[3] Tseng ML (2021). Sustainable industrial and operation engineering trends and challenges toward industry 4.0: A data driven analysis. J. Ind. Prod. Eng..

[4] Liu ZW (2017). Current situation, development demand and future trend of automotive technologies in China. Automot. Technol..

[5] Antonio OG, Jordi S, Roger S (2020). Sustainable European transport system in a 100% renewable economy. Sustainability.

[6] Li YX, Gao WJ, Ruan YG (2018). Grid load shifting and performance assessments of residential efficient energy technologies, a case study in Japan. Sustainability.

[7] Kuang X, Zhao FQ, Hao H (2019). Assessing the socioeconomic impacts of intelligent connected vehicles in China: A cost-benefit analysis. Sustainability.

[8] Chao PP, Ceng SY, Shen B (2013). Development analysis of China's automobile fuel consumption level and energy-saving technology. Auto Ind. Res..

[9] Wang XJ (2014). A study on the effects of the matching of automatic transmission on the energy consumption of electric vehicle. Automot. Eng..

[10] Eckert JJ, Santiciolli FM, Yamashita RY (2019). Fuzzy gear shifting control optimization to improve vehicle performance, fuel consumption and engine emissions. IET Control Theory Appl..

[11] Fu R, Zhang YL, Yuan W (2019). Progress and prospect in research on eco-driving. China J. Highway Transp..

[12] Avishai C (2021). Syncing sustainable urban mobility with public transit policy trends based on global data analysis. Sci. Rep..

[13] Óscar M (2020). An intelligent system-on-a-chip for a real-time assessment of fuel consumption to promote eco-driving. Appl. Sci..

[14] Huang W, Liu HJ (2018). Study on evaluation method of drivability quality for vehicle starting conditions. Automob. Eng..

[15] Chen SY, Hung YH, Wu CH (2016). An integrated optimal energy management/gear-shifting strategy for an electric continuously variable transmission hybrid powertrain using bacterial foraging algorithm. Math. Probl. Eng..

[16] Ma LY (2017). Bench test development of vehicle ride comfort based on road simulation. Lab. Res. Explor..

[17] Duque EL, Aquino PT (2015). Human factors analysis of manual gear shifting performance in passenger vehicles. Proc. Manuf..

[18] Huang H, Gühmann C (2018). Model-based gear-shift optimization for an automated manual transmission. Proc. Inst. Mech. Eng., Part D: J. Automob. Eng..

[19] Li H (2004). Research on Testing Bench for Automotive Transmission Assembly.

[20] Dong ZR, He P (2007). Design of bench test items for synthesis performance of vehicle automatic transmission. Automot. Ind..

[21] He YP, Rui YN, Liu S (2009). Research on the evaluation of gearbox design schemes based on extension methodology. Mach. Des. Res..

[22] Zhao ZX, Zhao LH, Ma J (2016). Application of shift quality assessment tools in practical work. Automob. Appl. Technol..

[23] Gao C, Ge WZ (2017). The optimization of the shift ability assessment in the heavy truck. Automob. Appl. Technol..

[24] Kramarz KD, Przybylska E (2020). Scenarios for the development of multimodal transport in the TRITIA cross-border area. Sustainability.

[25] Liu XJ, Sun DY (2020). An improved design of power-cycling hydrodynamic mechanical transmission. J. Mech. Sci. Technol..

